# Pharmacokinetics of the soluble guanylate cyclase stimulator riociguat in individuals with hepatic impairment

**DOI:** 10.1186/2050-6511-14-S1-P21

**Published:** 2013-08-29

**Authors:** Reiner Frey, Corina Becker, Sigrun Unger, Anja Schmidt, Georg Wensing, Wolfgang Mueck

**Affiliations:** 1 Clinical Pharmacology, Bayer Pharma AG, Pharma Research Centre, Wuppertal, Germany; 2 Global Biostatistics, Bayer Pharma AG, Pharma Research Centre, Wuppertal, Germany

## Background

Riociguat is the first oral, soluble guanylate cyclase stimulator currently under review for the treatment of pulmonary hypertension (PH), a progressive disease with high mortality [1-7]. The present pooled analysis assessed the pharmacokinetics of riociguat and its metabolite M1 (BAY 60-4552) in individuals with hepatic impairment (Child–Pugh A or B) and healthy controls. The safety and tolerability of riociguat were evaluated.

## Methods

Two non-randomized, non-blinded, observational studies with group stratification were included in the analysis. The studies were conducted in a single centre in Germany, in accordance with Good Clinical Practice and industry guidelines [8,9]. Individuals with liver cirrhosis (Child–Pugh A, n = 16; Child–Pugh B, n = 16) and 32 healthy age-, weight- and sex-matched volunteers received a single oral tablet dose of riociguat 1 mg. Dense sampling was performed for pharmacokinetic parameters.

## Results

**Figure 1:**
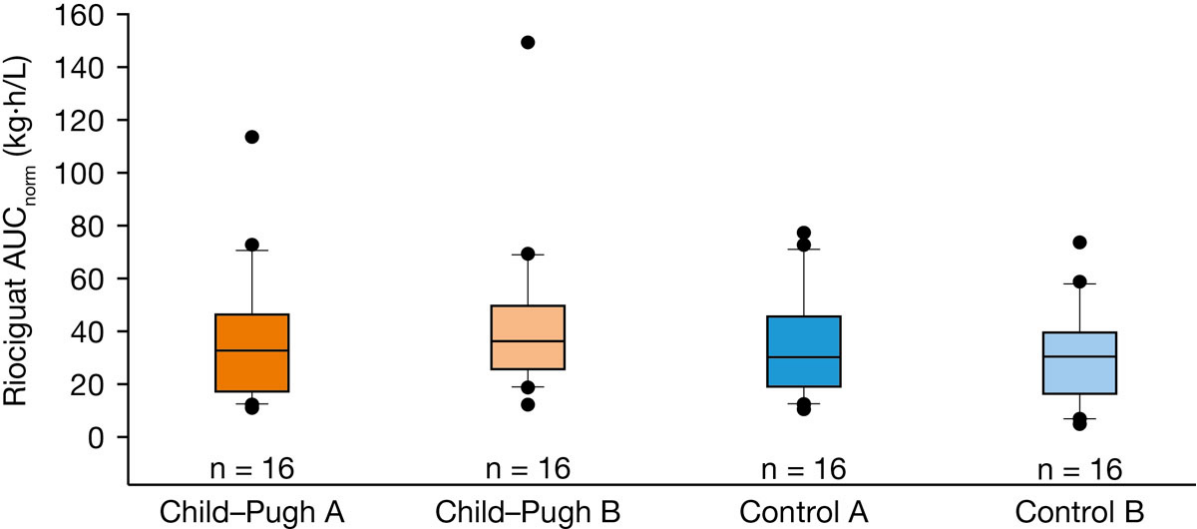
Box-and-whisker plot of riociguat AUC_norm_ (kg·h/L) after a single oral dose of riociguat 1 mg. Box, 25th–75th percentile; vertical line, 10th–90th percentile; horizontal line, median; more extreme values are plotted as points; individuals eligible for pharmacokinetic analysis, n = 64; AUC_norm_, area under the plasma concentration–time curve from time 0 to infinity divided by dose per kilogram of body weight for total riociguat.

Sixty-four participants (42 men and 22 women; mean age, 55.1 years [range, 35–72 years]) received riociguat and completed the study according to protocol. Owing to the rapid absorption of riociguat (median time to reach maximum concentration in plasma [C_max_], ≤ 1.5 hours in all groups), mean dose- and body-weight-normalized C_max_ values for total riociguat were similar in all groups (Table 1). Mean half-life of total riociguat was longer in the Child–Pugh B group than in the Child–Pugh A group and the controls (Table 1). Exposure (dose- and body-weight-normalized area under the plasma concentration–time curve [AUC_norm_]) to total riociguat was elevated in Child–Pugh B but not Child-Pugh A individuals compared with controls (Table 1, Figure 1). Antagonizing effects – reduced rate of formation and impaired M1 elimination – led to relatively small differences in overall exposure to M1 in the Child–Pugh A and B groups and their controls. Results for unbound riociguat and M1 were similar to those for total riociguat and M1. No serious or severe adverse events were reported. The most common drug-related adverse event was headache. There was no difference in safety or tolerability between study groups. Riociguat AUC and C_max_ ranges in patients with hepatic impairment overlapped those previously observed in healthy volunteers and patients with PH [2,3].

**Table 1:** Pharmacokinetic parameters of riociguat in plasma following a single oral dose of riociguat 1 mg

Parameter	Child–Pugh A (n = 16)	Child–Pugh B (n = 16)	Control A (n = 16)	Control B (n = 16)
AUC, μg·h/L	371.0 (74)	458.9 (62)	349.9 (67)	300.9 (92)
C_max,_ μg/L	42.67 (37)	43.27 (39)	42.67 (23)	38.68 (30)
AUC_norm_, kg·h/L	30.9 (75)	36.6 (65)	29.1 (67)	23.9 (94)
C_max,norm_, kg/L	3.56 (33)	3.45 (26)	3.55 (20)	3.07 (23)
t_½_, h	9.19 (53)	13.7 (50)	9.02 (63)	7.54 (86)

Values are geometric means (percentage coefficient of variation). AUC, area under the plasma concentration–time curve from time 0 to infinity; AUC_norm_, AUC divided by dose per kilogram of body weight for total riociguat; C_max_, maximum concentration in plasma; C_max,norm_, C_max_ divided by dose per kilogram of body weight for total riociguat; t_½_, terminal elimination half-life for total riociguat.

## Conclusion

Child–Pugh A individuals had similar plasma riociguat concentrations to controls. Child–Pugh B individuals had a higher exposure to riociguat than those in the other groups; particular care should be exercised during individual dose titration in patients with moderate hepatic impairment.
